# HPV Prevention series

**DOI:** 10.1186/1750-9378-7-37

**Published:** 2012-12-20

**Authors:** Silvia de Sanjosé

**Affiliations:** 1 Unit of Infections and Cancer, Cancer Epidemiology Research Programme, Institut Català d’Oncologia, Av. Gran Via de l’Hospitalet 199-203, 08908 L’Hospitalet de Llobregat, Barcelona, Spain

## Abstract

Human Papilloma Virus (HPV) is a major leading cause of Human Cancer. Through the HPV Prevention series we would like to highlight the quality and the breadth of the research being carried out on the Control and Prevention of HPV and HPV related disease. This series aims to bring together a diverse range of HPV related specialties featuring research that has as ultimate goal insights into HPV related disease reduction. Articles within a wide range of topics such as natural history studies, impact of screening interventions or impact of HPV vaccines will be most welcome.

## Editorial

Human Papillomavirus (HPV) is responsible for over five per cent of all cancers worldwide and of 15% of cancer in women in developing countries [1]. HPV is the necessary cause of cervical cancer [2], and is thought to be the necessary cause for anal cancer [3]. Cervical cancer is the third most common cancer in women worldwide with about 500,000 cases every year and remains to be the second leading cause of cancer death in women in less developed regions of the world [4]. HPV is also partially related to the etiology of vulva, vagina and penile cancers although these cancers are far less frequent, accounting for approximately 50,000 cases every year worldwide. Accumulated data also suggest a potential implication of HPV in oropharyngeal cancer among younger adults in wealthy populations [5]. Increasing trends in incidence are now being seen for anal cancer [6] and for oropharyngeal cancer [5] suggesting an increased background prevalence of oncogenic HPV types in affected populations. HPV is also the cause of genital warts, a non-malignant disease mainly related to HPV 6 and 11, that frequently affect sexually active men and women. Treatment of genital warts is tedious and not always effective but a dramatic descent in incidence has been shown in HPV vaccinated population [7].

Research on HPV and cancer has drawn a scenario in which a major reduction of HPV related disease is feasible through population based prevention strategies. These include routine HPV vaccination of pre-adolescent and young women, improvement of cervical cancer screening strategies through HPV based technologies, adaptation of preventive strategies using cost-benefit models and inclusion in the health priority lists the wide range of HPV related disease [8].

While our knowledge on the natural history and prevention mechanisms of cervical cancer has increased enormously during the last 30 years, there remain major unanswered issues [9]. A wide range of topics have been proposed and many of them will certainly be a priority for this HPV Prevention series. A question such as what is the role of micro RNA in HPV pathogenesis, why is the HPV 16 the most successful mucosotropic HPV type or how can advocacy deal with anti-vaccine activism are all relevant queries that can have a space in this collection if structured into research projects. It is clear that we need to open the focus of our research from cervical cancer to a much wider scope of HPV related diseases. Finally, ancillary research (i.e. management or associations with other STIs or educational approach to pre-adolescent health) can also be of interest in this series if linked to HPV related disease.

HPV related disease is not an entity that navigates on its own. It represents a complex mixture of genetics, micro-environment, behaviour and social influences. With the HPV Prevention series we hope to wider our minds, to increase our capacity for action and ultimately, to increase the health of our population.
